# Psychodynamic Based Equine—Assisted Psychotherapy in Adults with Intertwined Personality Problems and Traumatization: A Systematic Review

**DOI:** 10.3390/ijerph17165661

**Published:** 2020-08-05

**Authors:** Géza Kovács, Annemiek van Dijke, Marie-Jose Enders-Slegers

**Affiliations:** 1 Faculty of Psychology, Open University, Valkenburgerweg 177, 6419 AT Heerlen, The Netherlands; Marie-Jose.Enders@ou.nl; 2 SPEL Psychologen Putten, Garderenseweg 158, 3881 NE Putten, The Netherlands; 3 Online poli’s I-psy psyQ Brijder, Parnassia Group, Schipholpoort 20, 2034 MA Haarlem, The Netherlands; A.vanDijke@psyq.nl

**Keywords:** equine-assisted psychotherapy, psychodynamic psychotherapy, personality problems, traumatization, systematic review

## Abstract

The growing field of equine-assisted psychotherapy (EAP), a subfield of animal-assisted psychotherapy (AAP), needs theoretically-based clinical studies. This systematic review examines the existing clinical studies in adult populations on psychodynamic psychotherapy combined with equine-assisted psychotherapy. An electronic database search was divided in two studies to identify publications on 1) EAP combined with psychodynamic psychotherapy and 2) EAP combined to personality problems and traumatization in order to compile studies by population, intervention, outcome and therapeutic assets. Study 1 revealed no relevant clinical studies on EAP with a psychodynamic background with an adult population. Study 2 revealed 12 publications to review predominantly addressing veterans with PTSD. The methodological limitations of most of the studies restrain the overall findings on outcome. However, overall positive effects for EAP, specifically on its experiential features and on finding interpersonal trust for patients, can be discerned. There is an apparent need for clinical studies meeting methodological standards on psychodynamic underpinned EAP methodologies in adults with intertwined personality problems and traumatization.

## 1. Introduction

### 1.1. Psychodynamic Psychotherapy

Psychodynamic psychotherapy, both time-limited (short-term psychodynamic psychotherapy, STPP) and open-ended psychodynamic psychotherapy (PPT), is a well-researched treatment modality and applicable for various mental problems [1,2,3,4]. Psychodynamic psychotherapy is an intersubjective treatment in which the active ingredients are considered to be the enhancement of mentalization and internalization of a new attachment object through the interpretation of transference and countertransference experiences between therapist and patient [5]. Enhancing corrective emotional experiences processed in relation to the therapist entails much of the therapeutic work [5]. Corrective emotional experience is: “reexperiencing the old, unsettled conflict but with a new ending”. The emphasis is on the importance of working through painful emotional conflicts by experiencing new and more adaptive feelings within the therapeutic relationship. For example when the patient feels anger towards the therapist because the patient feels abandoned due to an upcoming vacation of the therapist, a corrective emotional experience could be that the patient feels comfortable to express his anger withstanding the risk from earlier experiences with an important other (usually the parents) that didn’t accept his anger and the patient was forced to an inadequate coping of the event.

The use of experiential interventions in order to acquire insight into the underlying patterns related to patients’ complaints from an interpersonal and intrapersonal perspective is prominent in the psychodynamic- and to the psychodynamic psychotherapy related schema-focused-psychotherapy [6]. Meta-analysis on psychodynamic psychotherapy [7] suggests equal efficacy to other psychotherapies, in addition Town et al. [8] found STPP had robust and persistent effects in patients with personality disorders.

### 1.2. Animal-Assisted Psychotherapy

Although seemingly different, animal assisted psychotherapy also focuses on interactions between individual patient, therapist and in this case animals. Worldwide, a development can be observed in which animal-assisted interventions can be distinguished within various forms of assistance [9]. In this paper we explore *Animal Assisted* (*Psycho)therapy* (AAP) as defined in the White Paper of The International Association of Human-Animal Interaction Organizations (IAHAIO; 2014). Animal-assisted psychotherapy conveys several human-animal interaction theories explaining the animal-human relationship. It includes biophilia- (emotional connection to other living beings), adult attachment- (a bi-directional connectivity between humans and animals) and social support theory (being cared for and loved, and being a member of a network of mutual obligations) in direct relation to the mutual, embodied attunement of behavior between humans and animals [10].

### 1.3. Equine-Assisted Psychotherapy

A subcategory of animal-assisted psychotherapy is equine-assisted psychotherapy (EAP) in which the horse serves as a supporter of psychotherapeutic interventions and as a mediator between psychotherapist and patient. The horse could be introduced in the psychotherapy as a non-verbal reciprocal transference- and transitional object in order to find corrective emotional experiences for the patient. A therapeutic triad is formed in which the animal supports the psychotherapeutic relationship and psychotherapeutic techniques for patients’ intra- and inter- psychological processes. Possible useful features of the horse are their social-relational aspects, sensory-intentional aspects, capability of carrying a person, their vulnerability and power. The explicit features of the horse could therefore elicit attachment—and mentalization themes on a cognitive-emotional—and bodily-emotional level next to influencing muscle-motor activity in patients, resembling ‘mother-child interactions’ [11]. Therefore, in EAP the horse functions as an intersubjective, accessible, safe Other (Other is used here in the psychodynamic meaning and therefore written with a capital) next to the psychotherapist through which the patient can learn to understand Others’ minds as well as their own [12,13]. EAP focuses beside the cognitive level more on the physical and emotional level by the nonverbal embodied hence experiential approach. Thus facilitating the psychotherapeutic process of the patient to learn to regulate affects and stress, to enhance self-direction and resilience, essential for instance in traumatized patients. [14,15,16].

The observable overlap between the targets proposed in psychodynamic psychotherapy and those in EAP might suggest that, since psychodynamic psychotherapy is well described and studied, it could form a fundamental theory for AAP including but not limited to EAP. Earlier conducted systematic reviews on animal-assisted therapy concluded that there is some evidence for the effectiveness of AAP but they call the need for comparative studies on AAP and other psychotherapies, studies that address the psychotherapeutic process in order to understand the working mechanism, and the development of theories on AAP [17,18,19,20,21].

The growing field of AAP needs questions answered about the efficacy and effectivity and on how the animal can assist the patient and the therapist during their therapeutic trajectory. To further the EAP-field, knowledge has to be shared with and about well-defined treatment methods based on a solid theoretical background. Therefore, we conducted a systematic review to examine the state of rigorous studies on psychodynamic-based equine-assisted therapies. This examination should be satisfied if a substantial amount of articles could be retrieved to shed light on psychotherapeutic assets and clinical relevance of equine-assisted psychotherapy in order to find relevant future directions for research.

## 2. Method

### 2.1. Existing Clinical Studies on Psychodynamic Based Equine-Assisted Psychotherapy

#### 2.1.1. Study 1: Method

An online search using MedLine and Pre-MedLine (PubMed alike), EMBASE and PsycINFO search systems was undertaken simultaneously, using the following terms: (equine assisted psychotherapy OR animal-assisted psychotherapy) AND (psychodynamic psychotherapy OR short-term psychodynamic psychotherapy). Search terms within each of the categories were linked with “or,” and larger categories were linked with “and” to capture studies that included at least one search term from all of the categories.

#### 2.1.2. Inclusion Criteria

Interventions or programs were included if they:

1) Were animal-assisted psychotherapeutic interventions with an emphasis on equine-assisted therapy with a psychodynamic characteristic for adults 18 years and over and currently experiencing symptoms or meeting criteria for a mental health condition.
2) Had been published (as journal articles) between 2015 and 2020.
3) Employed an experimental study design with a control or comparison condition, including randomized trials, matched group designs, and designs in which the sample served as their own control.
4) Included data on clinical effectiveness (e.g., changes in: interpersonal conflict and relationships, adaptive functioning, global functioning score, quality of life score, social skills, in stress response, ability to perform activities of daily living, ability to attend work/school/volunteering, changes in symptoms like PTSS symptoms, depression, mood, affect, illness perception).

#### 2.1.3. Exclusion Criteria

Studies were excluded if they did not have a specific focus on adult mental health, did not have a comparison or control group, or if the intervention did not involve animals (e.g., simulated or virtual animals).

**Figure 1:**
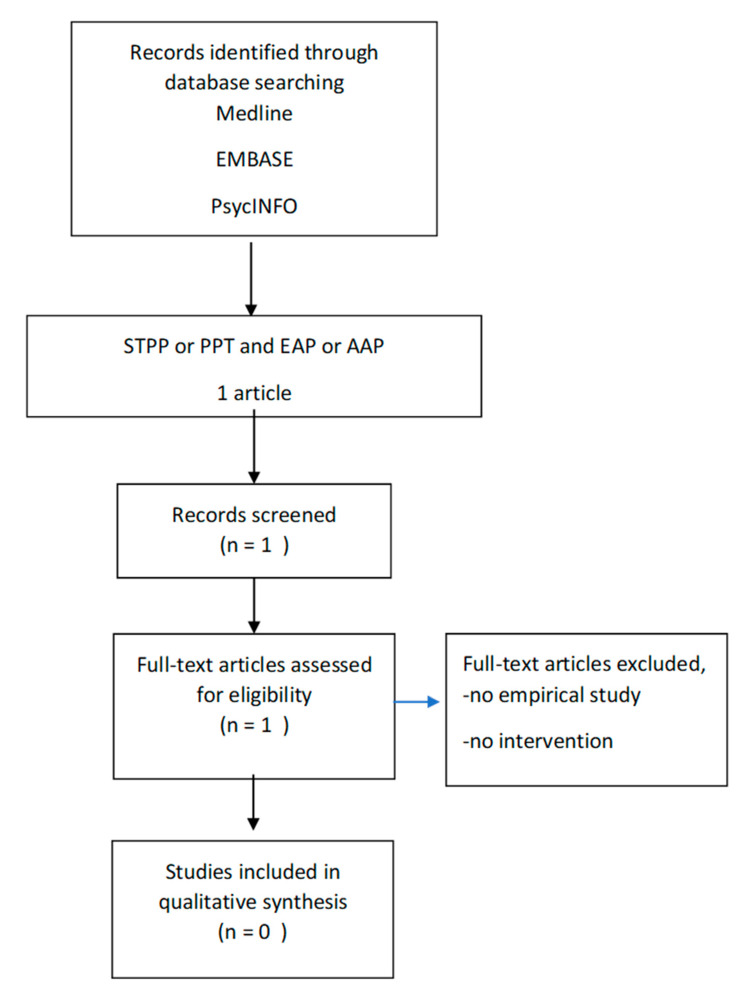
Flow diagram of the selection process.

See Figure 1 for the search history and results following the PRISMA paradigm [22]. Eligibility assessment was performed independently in unblinded standardized manner by 2 reviewers. The reviewers had to find consensus about the eligibility of the article. To summarize study outcomes, the results of each study will be subsequently organized by the most commonly reported outcomes, such as effect sizes, reported means and standard deviations.

## 3. Results

No studies were retrieved in this search on rigorous studies on (Short Term) Psychodynamic Psychotherapy combined with equine-assisted psychotherapy for patients in mental health care who underwent equine-assisted psychotherapies. In addition no studies were found in this search on rigorous studies on (Short Term) Psychodynamic Psychotherapy in general combined with animal-assisted psychotherapy for patient who underwent animal-assisted psychotherapy (AAP).

### 3.1. Existing Clinical Studies on Trauma and Personality in Relation to Equine-Assisted Psychotherapy

The lack of results in the first examination warrants a further exploration of the target population for EAP and psychodynamic psychotherapy. Psychotherapy indication for EAP is often related to patient reports of a history of psychological trauma and personality problems. 

#### 3.1.1. Study 2: Target Population

The proposed target population for psychodynamic psychotherapy and EAP, considering its overlap, are patients reporting Chronic Early Trauma (CET) in which trauma and personality problems are intertwined. CET means the harmful psychological, biological and social consequences of a combination of stressful and potentially traumatic events during childhood. These events are persistent, long-term, within the interpersonal context and with disruption of developmental stages resulting in unsafe attachment representations making it difficult for these patients to trust another person, for example a therapist. While to understand others’ minds as well as their own is important to process in psychotherapy for patients who suffered traumatization during the developmental years. The trauma reports have a linear relation to a complexity of commonly associated psychiatric disorders, ranging from attachment problems, (complex) posttraumatic stress disorder (PTSD), dissociative disorders, anxiety disorders, alcohol and drug addiction to depression, eating disorders, somatization disorders, schizophrenia/psychotic episodes and personality disorders (PD) e.g., [23,24,25,26,27,28]. Patients with personality disorders are characterized by a rigid pattern of internal experiences and behavior reflected on cognitive- and inter-personal functioning and on affect-and selfregulation abilities [29].

#### 3.1.2. Psychodynamic Based EAP with Intertwined Trauma and Personality Problems

The psychodynamic- and to the psychodynamic psychotherapy related schema-focused-psychotherapy are considered as most applicable for traumatized patients and PD patients. It has an intensive focus on attachment, problematic mentalization and relational aspects. To acquire insight into the underlying patterns related to patients’ complaints from an interpersonal- and intrapersonal perspective the use of experiential interventions is advocated [3,6,23]. The benefits of experiential interventions for PD patients [6] and the importance of embodied affect-and stress regulation for traumatized patients, among others namely introduced by Levine [30] and Porges [14], has its place in the psychodynamic approach. From this perspective equine-assisted psychotherapy could fit this paradigm. Several theoretical outlines of EAP have been published in which theoretical foundations are described with reference to psychodynamic-, trauma- attachment- and interpersonal focused treatment e.g., [11,13,31,32].

Therefore, in the second study we want to investigate the existing clinical studies on trauma reports/traumatization and personality problems in relation to equine-assisted psychotherapy to obtain the state of the amount of research, the populations involved, the form of the EAP intervention, outcomes and on the therapeutic assets and clinical relevance of equine-assisted psychotherapy. This examination should be satisfied if a substantial amount of articles could be retrieved to shed light on therapeutic assets and clinical relevance of studies on trauma reports/traumatization and personality problems in relation to equine-assisted psychotherapy in order to find relevant future directions for research.

### 3.2. Method

**Figure 2:**
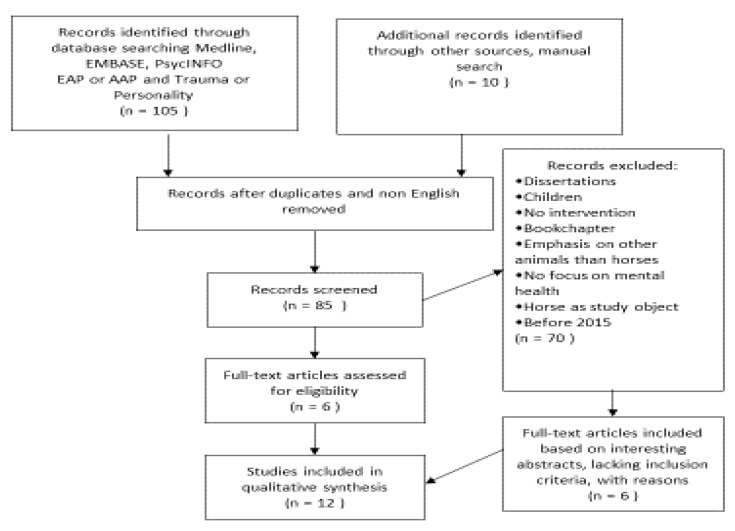
Flow diagram of the selection process.

An additional online search (see Figure 2) using Medline and Pre-Medline (PubMed alike), EMBASE and PsycINFO search systems was undertaken simultaneously, using the same in/exclusion criteria as in the first study and using the following terms: (animal-assisted psychotherapy OR equine-assisted psychotherapy) AND (Personality problems OR Traumatization). 

## 4. Results

We found two articles eligible for review [33,34]. A manual search revealed four more articles for review [35,36,37,38]. Due to their interesting abstracts we added six articles, five which lack control conditions [39,40,41,42,43] and one article involving adolescents [44]. Table 1 lists the 12 studies included in this review.

### 4.1. Population

This review encompasses seven articles (58%) with a patient populations of war veterans with PTSD [33,37,38,39,41,43], one article on pain patients [34], one article on women with social anxiety [35], one article on substance abuse [36], one article on traumatization in general [42] and one article on childhood traumatization in adolescents [44].

### 4.2. Design

Three studies (25%) incorporated a randomized control trial [33,34,35]. Three studies used a quasi-randomization to compare the experimental condition with TAU an EAP added or without EAP [36,38,44]. Six studies used a pre-post test design [37,39,40,41,42,43].

### 4.3. Intervention

In three studies horseback riding or therapeutic riding was involved [33,36,43]. Three studies described equine activities as the EAP condition [35,36,40]. One study used activities combined with mounted work [36]. Three studies explicitly mentioned a psychotherapy orientation, respectively CBT [35], Attachment-based psychotherapy [44] and Gestalt Therapy (here without psychodynamic reference to developmental issues) [37]. Three studies incorporated the Eagala model (no mounted work) [38,41,42]. Body-orientation and herd observation is mentioned in two articles [36,44]. The duration differed, five articles mentions a 6 week program [33,35,38,41,42], two articles described a 5 day program [37,40], one study used a 8 weeks program [43], in one study a 12 session program is described [36], one study investigated a 6 months weekly program [39] and one a 12 weekly 2 days program is mentioned [44].

### 4.4. Outcome

All articles mention a significant decline in symptoms. Out of the studies with a randomized control design one article elicits a significant decrease in PTSD symptoms [33], one article describing significant pain reduction [34], one article mentions a significant reduction of social anxiety [35] and one study found a significant stronger motivation to stay in therapy for substance abuse [36]. One study found no significant reduction in PTSD symptoms [38]. Significant increase of resilience was mentioned in three articles [38,41,44]. All the non-randomized, predominantly pre-post designed, studies found significant decrease in symptoms, except for one study who found no significant reduction of PTSD symptoms at 3 months follow up [37]. One study found a significant decrease of interpersonal sensitivity and phobic complains [40]. Two studies found a decrease in hyperarousal [40,44] and 1 study found significant improvements on vitality, social functioning and limitations due to emotional problems over time [43]. One article mentions no effect on respiration rate and systolic or diastolic blood pressure was found in war veterans with PTSD [40]. One study found no significant effect of the treatment on physical health, proactive coping, self-efficacy, social support, or life satisfaction [42].

### 4.5. Limitations

Overall, a low quantity of identified relevant articles was identified. Five of the total 12 articles incorporated a randomization and/or control condition, besides there was a lack of information on the long term effects. There was no blinding strategy involved in the selected articles. 

All studies had a relative small sample size, possibly influencing the external validity, except for the Kern et al. [36] study with N = 108. Most of the outcomes reported were based on subjective questionnaires which may create a risk for bias (in either direction depending on the perceptions and expectations of the participants and clinicians involved), except the studies by Burton et al. [38] who examined more objective items as cortisol and saliva and likewise Malinowski [40] on respiration rate and blood pressure. All studies reported *p*-values to indicate statistical significance, two studies by Balluerka et al. [44] and Lanning et al. [43] also reported effect sizes.

### 4.6. Clinical Relevance/Therapeutic Asset

All articles report relatively short programs whether or not as part of ongoing therapy trajectory. Four articles mention the bodily interaction [33,36,40,44], two in relation to horsebackriding [33,43] not EAP. Attachment and bonding with horses was mentioned by three studies [35,43,44]. One study mentioned the attachment between client and involved partner [37]. Providing safe-haven was reported by two articles [35,42]. The concept of trust/secure adult attachment and therapeutic alliance was mentioned in five articles [35,36,39,42,44]. The concept of resilience was mentioned by four articles [37,38,41,44]. The presence of a passive group of clients during EAP was mentioned in one study [42].

## 5. Discussion of Studies 1 and 2 in Unison

The growing field of AAP needs questions answered on *how* the animal assists the patient and the psychotherapist during their psychotherapy trajectory (mechanisms of change). EAP fits the psychodynamic psychotherapy realm conveying themes as insecure adult attachment, problematic mentalization, and experiential interventions considered important to be processed for psychologically traumatized persons. Therefore with this systematic review we wanted to investigate the existing studies with adult mental health patients who underwent equine-assisted psychotherapies with a psychodynamic approach. This examination was satisfactory if a substantial amount of articles could be retrieved to shed light on psychotherapeutic assets and clinical relevance of equine-assisted psychotherapy in order to find relevant future directions for research. This goal is not met.

### 5.1. Study 1

Our first study of this systematic review revealed no studies on EAP with a psychodynamic theoretical foundation. On one hand this could be considered fair in the light of the “Dodo-effect” considering the equal efficacy of all regular psychotherapies [45] implying an irrelevance of psychotherapeutic school, on the other hand it shows a gap in the light of the benefits of well described and researched methodologies, not in the least to train and inform psychotherapists (or other stakeholders) well [46,47].

### 5.2. Study 2

The second study, in which we reviewed clinical studies on adult patients on trauma and personality problems in relation to equine-assisted psychotherapy, revealed more eligible studies of which 50% incorporated a control condition. This examination was satisfactory if a substantial amount of articles could be retrieved to shed light on psychotherapeutic assets and clinical relevance of studies on trauma and personality problems in relation to equine-assisted psychotherapy in order to find relevant future directions for research. This goal is partially met. Much of the studies involved war veterans with PTSD of which it was unclear whether early childhood trauma was involved. Also no information on personality disorders or style could be retrieved. The assessment whether PTSD complaints are rooted in earlier developmental stages of the patient is important in order to define the route to recovery [25]. We expected more studies with an adult population related to early childhood trauma given the supposed utility of EAP for this group. The Balluerka et al. [44] and the Earles et al. [42] publications were the only ones who dealt with a population that could be considered CET patients, eliciting the need for more studies on EAP and CET population.

Although RCT designs are considered as the golden standard, also non-RCT-designs found in this review elicit useful research topics even if tentative conclusions, due to the study design, short programs or overall small sample size, are reported. Specially within the naturalistic social science and more so in researching animal-assisted psychotherapy blinding procedures run up against ethical issues, therefore other designs must be taken into account [48]. In line with the literature on psychotherapy research [48], also research on AAP should emanate from the complexity of clinical practice, which focuses on the psychotherapeutic process and is driven by theoretical concepts. In this context, more attention should go to single-case research, systematic observations, clinical experience and subjective changes in the patient, because it is precisely this the clinician is working with.

Also in the second study no articles align with/match the psychodynamic psychotherapy realm, except for the article by Balluerka et al. [44] although it dealt with adolescents. In this study attachment-based theory was combined to EAP explicitly in which inclusion of the horse complemented the attachment-based approach [44]. An inclusion criterion in this review was an adult population, while more studies on AAP and EAP with a population under 18 years of age were available. It would be interesting to examine the effect of AAP and EAP on the early onset of traumatization and personality problems.

Overall the studies didn’t show a clear rationale on how and why the horse is included in the psychotherapy and in some studies one could question whether the intervention could be defined as psychotherapy.

Remarkable is the relative short duration/low number of sessions of most of the interventions and with that it is unclear how and if the EAP is embedded in an ongoing treatment trajectory. Since CET has persistent and long-term consequences one could not assume that for example a 6 weeks program could elicit adequate recovery for these patients. The results of these very short interventions [33,35,36,37,38,40,41,42] could only be interpreted as a possible positive impulse for recovery.

The review did show the bodily interaction, physicality and bonding with the horse, possibly enhancing trust and the psychotherapeutic alliance, as psychotherapeutic assets [33,36,40,44]. In line with a study by Bamelis et al. [6] who found the involvement of experiential techniques highly beneficial in psychotherapy for PD, the benefits of equine-assisted psychotherapy could lie in the embodied experiences which relate to the attachment theory. The severe consequences of adverse childhood experiences on mental health patients [26,49] and for some possible insufficient alignment to the more cognitively oriented (and protocol-based) therapy methods [50,51,52], justify the expansion of treatment modalities. Different patients may benefit from different approaches or may benefit through different routes [53], specifically for adults with insecure attachment with traumatization originating in early (preverbal) development.

With due observance of the limitations of the retrieved studies, a positive trend in outcomes in favor of EAP can still be observed. Equine-assisted psychotherapy could be an applicable alternative for hard to reach patients considering its therapeutic asset on trusting others and establishing a fruitful psychotherapeutic alliance as several studies in this review mentioned [35,36,39,42,44].

For instance, Luyten et al. [1] state that ruptures in the psychotherapeutic alliance are inevitable with all patients, and that it is the extent to which both psychotherapist and patient manage these disruptions which relate to psychotherapeutic outcome. The challenge here is to restore epistemic trust in which experiences of interpersonal relatedness (mutuality and understanding) and self-definition (separateness) synergistically interact in a mutual facilitating process [1]. The inclusion of an animal in the psychotherapeutic process could help the restoring of this epistemic trust, crucial for therapeutic change [2]. Restored epistemic trust helps the patient to reflect in different ways about him-her self and others, opening the patient up to a social learning process outside the consulting room, possible with the perceived non-judgmental animal as a means of practice in relation to the psychotherapist and to the outside world.

### 5.3. Limitations and Strengths

There are some limitations to be addressed in this review. First, due to the remarkably zero retrieved studies in the first study, which urged us to add a second search, it might have led to a heterogeneous search. Although the addressed search items can be considered to be interrelated. Secondly, the search was restricted to English-language publications, it might be that eligible studies could be found in other languages. Thirdly, one has to consider the possibility of a publication bias due to the fact that clinical trials are more likely to be published if the results are statistically significant, omitting crucial information on non-significant results. Applicability of this review might be affected by the stringent inclusion criteria which did not deliver many results. However, adding an additional search with EAP and/or AAP and/or trauma and/or personality brought up studies with implicit psychodynamic key-aspects shedding light on therapeutic assets and clinical relevance of equine-assisted psychotherapy. Furthermore, one could consider the strength of this review lying in the fact that it clearly shows the lack of relevant efficacy studies combining psychodynamic- and equine-assisted psychotherapy.

## 6. Conclusions and Recommendations

The conclusions are two-fold. Firstly, in this rather new field of equine-assisted psychotherapy there are only a few published studies. Most of the literature about equine-assisted psychotherapy reports about therapies of all kind (by physiotherapists, nurses, social workers, psychologists) however seldom about equine-assisted psychodynamic psychotherapies. It shows that the AAP and EAP field is still in its infancy, so it is understandable that there are no relevant efficacy studies published combining psychodynamic- and equine-assisted psychotherapy between 2015 and 2020 for adult patients.

Therefore, in line with earlier systematic reviews on animal-assisted psychotherapy the expressed need for theoretical underpinned methodologies studied with comparative designs is still apparent. The body on evidence on the existing (classical) psychotherapies should form a basis for research in the field of animal-assisted psychotherapy. We consider animal-assisted psychotherapy as an (add-on) intervention building on the knowledge of the existing classical psychotherapy models. Especially considering the advances in neurobiology connected to psychoanalytical thinking, among others studied by Solms [54], studies on equine-assisted psychotherapy could contribute to this line of research due to the imposed non-verbal self-regulation strategies [11]. Consistent with other studies on the mechanisms of change in psychotherapy [47], which findings suggest that adherence (and competency) to technique and the psychotherapeutic alliance are interdependent and interrelated in predicting the success of psychotherapy, the establishing of a clear base (here a psychodynamic approach) is necessary. It helps the psychotherapist to be able to carefully scrutinize when, why and how specific techniques can be utilized in clinical practice [46]. For that matter also other theoretical-evidenced based psychotherapies, such as cognitive-behaviour therapy, complemented by animal-assisted psychotherapy could elicit the development of more personalized forms of interventions.

Secondly, this review shows that the experiential character of equine-assisted psychotherapy could be a beneficial factor for patients with intertwined trauma and personality problems. Possibly, EAP is influencing trust and the psychotherapeutic alliance positively. Also, stronger motivation to stay in psychotherapy and increase of resilience was associated with EAP. This is relevant as patients with intertwined trauma and personality problems tend to drop out of psychotherapy and have low expectations of treatment.

### Future Directions

In order to establish EAP as a feasible treatment modality for vulnerable patients, like CET and to further the field of AAP and EAP, we suggest five future directions. Firstly, as already mentioned, establishing a clear outline of procedures of an integrated equine-assisted psychodynamic orientation in clinical studies. A proposed outline should avoid a protocol-based approach but should preferably enhance the personal approach to psychotherapy. Secondly, to conduct empirical clinical studies comparing classical psychodynamic orientated psychotherapy to an integrated equine-assisted psychodynamic psychotherapy for CET patients. Thirdly, focusing on the mechanism of change from a psychodynamic- and attachment-based view like establishing epistemic trust, embodied affect- and stress regulation, inter-and intrapersonal deficits such as self-efficacy and resilience in traumatized patients as several reviewed studies elicited. Fourthly, next to quantitative studies, qualitative studies, whether or not as part of a mixed model, could draw insight in mechanisms of change from a patient perspective. Fifthly, we recommend to gain insight in the personality characteristics of patients who choose EAP allowing for a more transdiagnostic approach of comorbid symptomatology in order to explore ‘what works for whom’ [46,55].

## Figures and Tables

**Table 1:** Articles included in the review.

	Journal	Population	Design	Intervention	Outcome and Limitation	Clinical Relevance/ Therapeutic Asset
1. Alfonso, L., Llabre, M., Fernandez, I. 2015 [35]	*The Journal of Science and Healing*, 11/2015, V. 11, 6	Social anxiety in young women, N = 12	Randomly assigned to experimental or a no-treatment condition. Pre-post and follow up.	combining equine-assisted activities and cognitive-behavioral strategies to reduce symptoms of social anxiety	Experimental condition significantly greater reductions in social anxiety scores from baseline to immediate-post and from baseline to follow-up.	-success experiences -teambuilding -bonding with horse -trust in others -safe haven to explore emotions
2. Balluerka, N., Nekane, M., Muela, C., 2015 [44]	*Child Abuse & Neglect*, 2015, V.50 2015, pp. 193-205.	adolescents in residential care suffered traumatic childhood experiences with mental health problems. N = 63	Sequential two-armed design, N = 39(19 female and 20 male; mean age = 15.03) EAP+ TAU and a control group of 5 female and 19 male; mean age = 15.67 TAU	EAP involves an ongoing treatment with clearly established goals, 12 weeks of 2 days consecutive. Attachment-based psychotherapy combined to EAP. Six thematic blocks: (1) establishing a secure base, (2) identification, understanding and verbalization of emotions, (3) emotional regulation, (4) Interpersonal relationships, (5) self-esteem and self-competence, and (6) close.	EAP compared TAU: sign.reduction hyperactivity, large ES; adaptive skills sign improved, large ES; Sign.improved social adaption, moderate ES. Pre-post EAP condition: sign reduction symptoms and depression, resp. moderate and large ES; improved social skills, large ES; Interpersonal and self-esteem, moderate ES -no randomization, sequential assignment -relatively small N	-secure attachment, relations -coping skills - touching and being touched: reciprocity and synchrony - The horse as a mirror of emotions and behaviors - Response to success and failure, resilience -six clear blocks (structure) - internal working models and defense mechanisms. - the natural environment generated a sense of security -presence of other animals (i.e., dogs)
3. Bolden L.; Bentley D.; Adkins S.; Jagielski C.; Schwebel D, 2017 [34]	*Archives of Physical Medicine and Rehabilitation. 94th Annual Conference of the American Congress of Rehabilitation Medicine,* ACRM 2017. USA. 98 (10) (pp e117), 2017.	Perceived pain of patients with spinal cord injury (SCI). N = 25	A randomized control trial. Experimental condition *n* = 11	No information on the intervention	the average pre to post NRS score decreased for participants in the intervention group, but increased for participants in the control group	No information on the intervention
4.Burton L., Burge, M., 2015 [41]	*Journal of Investigative Medicine*. Conference: American Federation for Medical Research Western Regional Meeting, AFMR 2015. Carmel, CA USA, 63 (1) (pp 165), 2015	War veterans, N = 10 with PTSD	Pre-post, 6 weeks design; PTSD and resilience	6 weeks EAGALA sessions, no riding	Reduced PTSD-related symptoms and increased adaptive coping skill of Resilience. -no control -small N	-brief intervention reduces symptoms -no information on long term effects
5. Burton, L., Qeadan, F., Burge M., 2019 [38]	*Journal of Integrated Medicine* 2019;17(1):1419	War veterans, N = 20; experimental condition *n* = 10, control *n* = 10	a sequentially assigned, two-arm parallel group trial comparing 6 weeks of EAP with standard, previously established, ongoing PTSD therapy	Eagala; group; ground-work focusing on metaphors an awareness of emotions	Significant decrease PTSD, increase resilience both groups. No significant difference in PTSD, resilience and salivary cortisol compared to control. Low N, no information on control condition, short program	-decrease PTSD symptoms -increase resilience -experiencing stress relieve and improved self esteem -use of metaphors -use of a group
6. Earles, J., Vernon, L., Yetz, J., 2015 [42]	*Journal of Traumatic Stress*. Vol.28(2), 2015, pp. 149-152	PTSD symptoms following accident, physical or sexual assault, life-threatening illness or injury and sudden, violent death. N = 16	Pre-post design; 6 weekly 2-h sessions measuring: Posttraumatic stress; Trauma emotion; Generalized anxiety; Depression; Alcohol use; Physical health; Mindfulness; Proactive coping; Self-efficacy; Social support; Life satisfaction; Optimism	Eagala, no riding; individual therapy in group	PTSD symptoms, emotional distress, anxiety symptoms, depression symptoms, and alcohol use decreased significantly, increase in mindfulness. No significant changes in physical health, proactive coping, general perceived self-efficacy, social support, life satisfaction, or optimism. -no control -no follow up	-less intensive outpatient program potential to be effective in reducing symptoms of anxiety and depression. -passive group might have effects -non verbal, mindfulness elements, creating safe place, setting boundaries and non critical self-awareness might be important ingredients
7. Johnson et al., 2018 [33]	*Military Medical Research.* 5(1):3, 2018	veterans diagnosed with (PTSD) and/or traumatic brain injury (TBI), N = 29	randomized waiting-list (resp. *n* = 15, *n* = 14) controlled design with repeated measures	a 6-week therapeutic horseback riding (THR) program for decreasing PTSD symptoms and increasing coping self-efficacy, emotion regulation, social and emotional loneliness	significant decrease in PTSD scores and loneliness. Coping, self-efficacy, emotion regulation trended to improve. Outcome effects caused by longer program. -low N -short intervention, no follow up-not clear to be considered as psychotherapy	-less PTSS -bodily interaction with horse -self efficacy likely to improve
8. Kern-Godal, A., Arnevik, E., Walderhaug, E., Ravndal, E., 2015 [36]	*Addiction science & clinical practice*, 10/2015, V. 10, 1	young hospitalized substance users N = 107	An intention-to-treat design, to compare treatment as usual (*n* = 43) with treatment as usual plus HAT (*n* = 65).	EAT as complementary to TAU. Activity and mounted work, herd observation and body-oriented	EAT supports motivation to stay longer in treatment	-therapeutic alliance -different environment -physical activity -individual attention -comorbidity
9. Lanning, B., Wilson, A., Krenek, N., Alexander Beaujean, A., 2017 [43]	*Occupational Therapy in Mental Health*, 2017, V. 33, 3	War veterans with PTSD, N = 51	Pre-post design N = 51; 8-week therapeutic riding program	therapeutic riding program	significant decrease in PTSD symptoms with large ES, improved social functioning, vitality, less interference of emotions on daily activities, and increased participation with large ES. -no control -no follow up	-interaction with horse -participation in activities
10. Malinowski, K. et al. 2018 [40]	*Journal of Equine Veterinary Science*. 64:17-26, 2018	PTSD in Veterans. N = 7	Pre-post design	5 days EAA, involves equine activities	BSI inventory significantly reduced except for interpersonal sensitivity and phobic anxiety. Significant reductions Hyperarousal Symptoms of PTSD. HR was significantly reduced on day 2 involving grooming and petting in contrast to more physical activity other days. No effect on respiration rate and systolic or diastolic blood pressure.	-no information about the intervention. -short program -activities is not therapy -grooming reduced heart rate
11. Romaniuk, Evans, Kidd, 2018 [37]	*PLOS ONE*, 09/2018, V.13, 9	Defense Force veterans and their partners; domains of depression, anxiety, stress, PTSD, happiness, quality of life. N = 47	A non-controlled, within-subjects longitudinal design (pre- post-interven-tion and 3 months follow-up). Individual and Couples programs between subjects comparison	equine-assisted therapy (non riding) in residential program of 5 days Relational Gestalt Therapy: exploration of issues, challenges, and behaviours and awareness of responses e.g., fear, anxiety, danger.	equine-assisted therapy useful in the reduction of depression, anxiety, stress, PTSD symptoms and the improvement of happiness and quality of life. Gains short-term unless partners are integrated into the intervention. -no explanation about the effects -no control -small N -short program	-reduction of symptoms short term -partners’ secure attachment conveys a form of resilience in adversity. -intervention without explanation/processing developmental issues might influence sustainability of effects.
12. Shelef, A., Brafman, D., Rosing, T., Weizman, A., Stryjer, R., Barak Y., 2019 [39]	*Military Medicine*, 2019, 06	Patients with PTSD (veterans) N = 13; measuring PTSD on daily functioning and work	open case series pre-post design, 6 months	Riding and groundwork group therapy, weekly 3 months.	significant improvement in daily functioning and work after 6 months. -no control -low N	-improvement in performing daily tasks and work/study. -locus of control and trust through structured exercises in relation to the horse.
